# Taking leadership over psychopathogenic environments

**DOI:** 10.1192/bjo.2021.653

**Published:** 2021-06-18

**Authors:** Alastair Cockburn, Jane Morris

**Affiliations:** 1 Royal Infirmary of Edinburgh; 2 Royal Cornhill Hospital

## Abstract

**Aims:**

Do psychiatrists believe children are growing up in psychopathogenic environments that significantly contribute to mental ill- health? If so, do they feel empowered to change those environments? If not, how can psychiatrists be given a role where they can create meaningful change? Finally, how much responsibility can psychiatry usefully take for changing psychopathogenic environments?

**Background:**

We define psychopathogenic environments as environments that predispose to mental ill-health. It is the psychological environment we live in - including income, the way we interact with others (e.g. social media, bullying), what we do with our free time, pressures at school and expectations of our peers. It is not discrete events (e.g. trauma) and stretches beyond life at home (where many ACE's occur).

Self-harm presentations to medical professionals amongst teenagers are on the rise, Universities report a fivefold increase in disclosure of mental health conditions in the last decade. Here we consider if psychopathogenic environments are part of the cause of these changes.

**Method:**

A 10-item questionnaire distributed to Child and Adolescent Psychiatrists in NHS Lothian, NHS Grampian and Manchester University NHS Trust via a consultant in each Trust.

**Result:**

All 14 respondents said psychopathogenic environments are “very important” contributors to mental ill-health. 13/14 say the environments have got worse in the last 10 years. 13/14 responded negatively about whether psychiatrists could change them. When given white space to tackle the problem they suggested changes were needed from Government including against poverty / inequality, education, public health nudges, more resources, MDT working and better access to leisure facilities. Given specific choices, 11/14 identified influencing Government as a major way forward.

**Conclusion:**

This group of psychiatrists believe psychopathogenic environments are; 1) a very important contributor to mental ill-health 2) getting worse but 3) feel largely powerless to tackle it. It is a problem they think is important and want to engage in, but lack time, resources and struggle with the complexity of the problem. How therefore, can psychiatrist show leadership in this area? The two perspectives to consider how to empower psychiatrists to help create change are 1) how they can influence the environment for individual patients, 2) how they can influence public policy and government to make wider changes.

Is this the job of psychiatrists? Not alone, but as agents they have a unique insight and authority as both a lens for and director of these environments.

